# Hydrogen atoms in protein structures: high-resolution X-ray diffraction structure of the DFPase

**DOI:** 10.1186/1756-0500-6-308

**Published:** 2013-08-02

**Authors:** Mikael Elias, Dorothee Liebschner, Jurgen Koepke, Claude Lecomte, Benoit Guillot, Christian Jelsch, Eric Chabriere

**Affiliations:** 1 Weizmann Institute of Science, Biological Chemistry, Rehovot, Israel; 2 Cristallographie Résonance Magnétique et Modélisations, CNRS UMR 7036 Université de Lorraine, Vandoeuvre-lès-Nancy, France; 3 Max-Planck-Inst. of Biophysics, Department of Molecular Membrane Biol., Frankfurt/Main, Germany; 4 Unité de Recherche sur les Maladies Infectieuses et Tropicales Emergentes, IRD/CNRS, Université de la Méditerranée (Aix-Marseille II), 13 385 Marseille, France

**Keywords:** Sub-Ångstrom X-ray crystallography, Hydrogen atoms, DFPase, *MoPro* software

## Abstract

**Background:**

Hydrogen atoms represent about half of the total number of atoms in proteins and are often involved in substrate recognition and catalysis. Unfortunately, X-ray protein crystallography at usual resolution fails to access directly their positioning, mainly because light atoms display weak contributions to diffraction. However, sub-Ångstrom diffraction data, careful modeling and a proper refinement strategy can allow the positioning of a significant part of hydrogen atoms.

**Results:**

A comprehensive study on the X-ray structure of the diisopropyl-fluorophosphatase (DFPase) was performed, and the hydrogen atoms were modeled, including those of solvent molecules. This model was compared to the available neutron structure of DFPase, and differences in the protein and the active site solvation were noticed.

**Conclusions:**

A further examination of the DFPase X-ray structure provides substantial evidence about the presence of an activated water molecule that may constitute an interesting piece of information as regard to the enzymatic hydrolysis mechanism.

## Background

Organophosphates (OPs) are potent toxic compounds that inhibit acetylcholinesterase, a key enzyme within the central nervous system. These compounds have been extensively used as agricultural pesticides, while their toxic properties have fostered the development of chemical warfare agents such as soman, sarin and VX [1]. Since current methods implemented to remove such compounds prove slow, expensive and generate environmental concerns as well [e.g.; bleach treatment and incineration], enzymes that are capable of detoxifying OPs prove therefore particularly valuable [2].

Several different protein families have been described as OPs-hydrolases. For instance, microbial phosphotriesterases (PTEs) hydrolyze these compounds with high efficiency, close to the diffusion limit against some OPs, as demonstrated for the PTE from *Pseudomonas diminuta*[3]. A closely related protein family was recently discovered and labeled Phosphotriesterase–like Lactonase (PLL) [4]. Although its members are natural lactonases which could be involved in the regulation of the quorum sensing [5,6], these enzymes possess a promiscuous activity against a broad range of phosphotriesters [7,8]. In Human, the High Density Lipoprotein (HDL) associated human paraoxonase-1 is also capable of hydrolyzing OPs. The structure of a chimeric mammalian recombinant paraoxonase-1 was solved, and displayed a six-bladed *ß*-propeller with two calcium ions in a central water-filled tunnel [9]. The diisopropyl fluorophosphatase (DFPase) from the squid *Loligo vulgaris* is able to detoxify a broad range of organophosphorous compounds, including tabun, sarin and soman although its favorite substrate remains the diisopropyl fluorophosphate [10]. The DFPase is a 35 kDa calcium-dependent enzyme that exhibits a six-bladed *ß*-propeller structure with two calcium ions in a central water-filled tunnel, similar to that of the mammalian recombinant paraoxonase-1 [11].

The catalytic mechanism of DFPase has been investigated. Blum *et al*. [12] elicited the following mechanism based on a H_2_^18^O isotope labeling experiment: the calcium-coordinated Asp_229 was identified as the nucleophile that attacks the phosphorus center of the substrate via the formation of a phosphoenzyme intermediate. The enzyme would then be regenerated by a solvent water molecule that was not yet identified. Another possible catalytic mechanism for DFPase might involve the activation of a water molecule, as in PTEs [13], PLLs [6], human paraoxonase-1 [14], and the lactonase from *Staphylococcus aureus* (drug resistance protein 35) [15], a DFPase structural homologue. Under such conditions, it is the reactive hydroxide ion which acts as a nucleophile, attacking the phosphorus center of the substrates.

The experimental determination of the protonation states of amino acids in the active site pocket would help to discriminate between both mechanisms. The sub-Ångstrom resolution X-ray (0.85Å) structure was previously determined [11]. Congruently, Blum *et al.*, (2009) have performed a neutron diffraction study at 2.2 Å resolution, benefiting thereby from the high diffraction power of the DFPase crystals [16] and obtained a combined neutron-X-ray (X-N) model. Since both sub-Ångstrom X-ray resolution data and medium resolution neutron data are available for DFPase, the respective structures enables cross-comparison between hydrogen bonds and water networks. In our report hereby, we provide a detailed account regarding the water molecule protonation states within the DFPase active site.

## Results

### Hydrogen atoms in electron density

**Figure 1:**
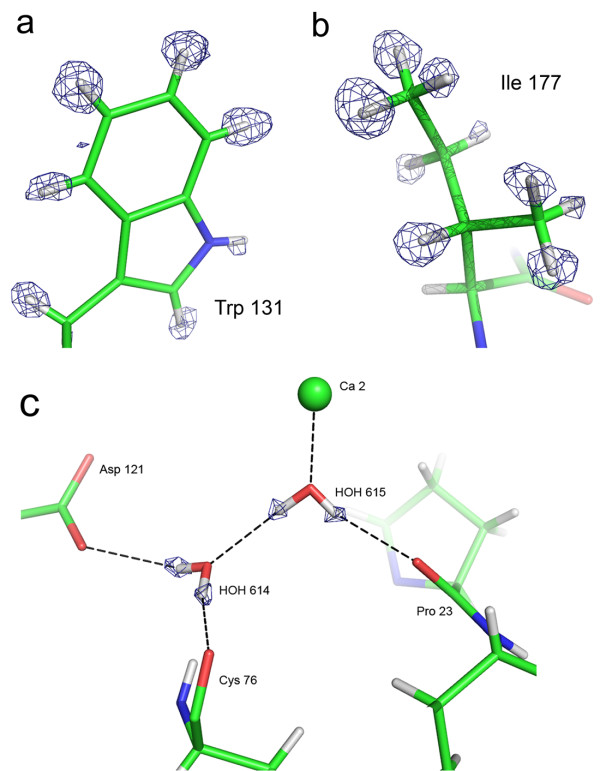
**View of hydrogen atoms in the X-ray structure. (a)** Fixed H atoms: Trp 131 side-chain. **(b)** Mobile hydrogen atoms: Ile 177. **(c)** Water molecules: water 614 and 615 and their hydrogen bond network. *F*_obs_-*F*_calc_ omit map in blue. The contour level is 2.6σ in **a)**, 2.7σ in **b)** and 2.5σ in **c)**. Hydrogen atoms have been omitted in the structure factor calculation of the maps.

After refinement, the *R* and *R*_free_ factors of the DFPase X-ray model improved from 11.1 and 12.8% initially [17], down to 10.3 and 12.1%, respectively. In the electron density maps, a significant part of the hydrogen atoms, --namely in low thermal motion regions--, appears clearly as electron density peaks in *F*_obs_-*F*_calc_ omit maps (Figure 1a and b), as in other sub-Ångstrom X-ray structures, such as Aldose Reductase [18] or PfluDING [19]. Thus, the position of numerous hydrogen atoms can be determined experimentally. Even more significant, the hydrogen atoms of several water molecules in the vicinity of the active site displayed most significant electron density peaks, up to 4.2 σ contour level. Out of the 481 water molecules in the DFPase structure, the positions of hydrogen atoms of 20 water molecules could be established solely on account of electron density peaks (Additional file 1: Table S1). It was unnecessary therefore to take into account explicitly the chemical environment in order to build the hydrogen-bond network (Figure 1c). The small fraction of water molecules for which hydrogen atoms are visible interestingly comprise active site water molecules, thereby allowing us to construct unequivocally, the hydration shell and the water hydrogen-bond network in the DFPase central tunnel.

### Water hydrogen bonds comparison

**Figure 2:**
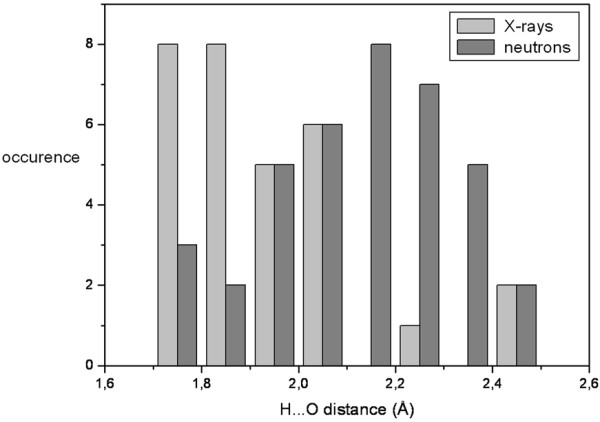
**Bar plot of the donor H…O distances in the X-N and X-Ray structure water H-bond network.** The mean values are 2.12 (rmsd = 0.19) Å and 1.93 (rmsd = 0.18) for neutrons and X-rays, respectively. The mean H-bonding distance for water molecules in the X-ray structure is significantly shorter than for the X-N structure.

Differences appear while comparing the DFPase X-ray and X-N structures (PDB ID 3BYC). In particular, the solvation can be different. For example, the orientation of some water molecules differs significantly (notably: DOD_1010, DOD_1052 and DOD_1066). Moreover, in some cases, the hydrogen bond partners are not identical, despite similar oxygen atom positions. In order to further quantify such findings, we carried out an analysis of the hydrogen bond geometry. As a result, for H-bonds between water hydrogen (H_w_) and protein or water oxygen atoms, the H_w_…O distances are shorter in the X-ray structure compared to the X-N model (Figure 2). This is not due to the intrinsic differences between the two techniques, as the O-H_w_ distances were normalized in the X-ray structure to the standard neutron diffraction value (see Methods). The average of thirty H_w_…O distances in the X-ray structure reaches 1.93 Å (rmsd = 0.18 Å), whereas it amounts to 2.12 Å (rmsd = 0.19 Å) for the 38 hydrogen bonds in the X-N structure.

**Figure 3:**
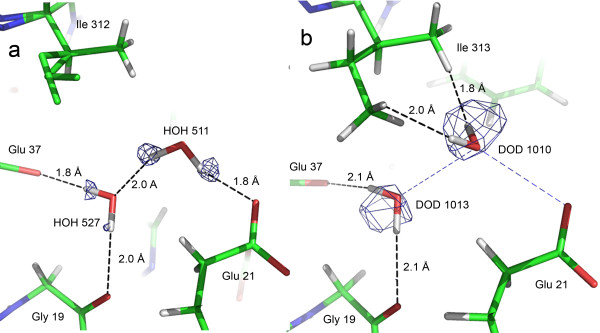
**Water molecule discrepancies between the sub-Ångstrom X-ray structure and the X-N structure.** View of **(a)** HOH 511 (X-ray structure) and **(b)** DOD 1010 (X-N structure), which are equivalent. An electron density *F*_obs_-*F*_calc_ omit map at 2.4σ contour level in **(a)** and a nuclear 2*F*_obs_-*F*_calc_ map at 1.4σ contour level in **(b)** are superposed to the structure. For comparison, the H-bonding of HOH 511 in the X-ray structure is indicated as blue dashed lines in **(b)**. Hydrogen atoms of the disordered part of Ile 312 in **a)** were not placed in the X-ray structure.

Interestingly, the average H_w_…O distance in the X-N structure is significantly longer. It is noteworthy that there are several short contacts between water hydrogen / deuterium atoms and neighboring hydrogen (or deuterium) atoms in the X-N model (59 interactions for which the H_w_…H or H_w_…H_w_ distance is below 2 Å). For instance, water molecule 511 (X-ray structure numbering) illustrates the differences found between the X-ray and X-N structures (Figure 3). The HOH_511 hydrogen atoms point each towards a neighboring oxygen atom (a glutamate and a water molecule) and clearly form hydrogen bonds. On the other hand, the two deuterium atoms of the corresponding water molecule in the X-N structure, DOD_1010, are not involved in any clear hydrogen bond.

### Active site water

As stated here-above, the orientation of numerous water molecules differs in the X-N and the X-ray structure. Particularly, the active site DOD_1033 is interesting since this molecule coordinates the catalytic calcium cation. Its configuration is unusual, as one of its deuterium atoms points towards the calcium ion with a short contact distance (H_w_…Ca2+ distance: 2.0 Å, O-H_w_…Ca2+ angle: 111.9°, see Figure five in [20]. As these atoms (H and Ca2+) are both positively charged, the configuration is rather unfavorable.

**Figure 4:**
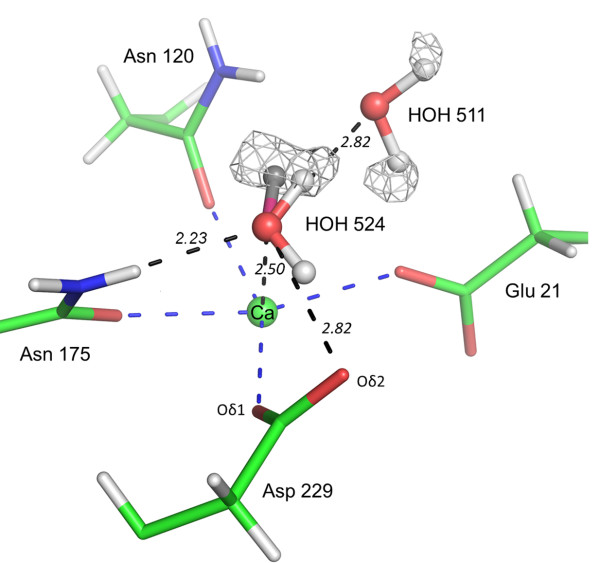
**Chemical environment of calcium coordinating water molecule HOH 524.** The electron density F_obs_-F_calc_ residual map (light grey) is at 2.2 σ contour level. The two possible protonation states of HOH 524 are modelled (protonated form in light grey (hydrogen) and red (oxygen); deprotonated activated form in grey (hydrogen) and pink (oxygen)). The interactions performed by HOH 524 are shown as black dashed lines. The calcium coordination is highlighted by blue dashed lines. Distances are indicated in Ångstrom.

In the X-ray structure, a strong electron density peak (maximum contour: 4σ) is visible close to the equivalent water molecule (HOH_524), which can be unambiguously allocated to one of its hydrogen atom (Figure 4). Moreover, this electron density peak displays an elliptical shape, whereas all the other electron density peaks revealing water hydrogen atoms in the DFPase X-ray structure are spherical.

The analysis of the chemical environment of the water molecule in the X-ray structure shows that HOH_524 is involved in the following interactions (Figure 4): (i) Its hydrogen atom, located with a strong electron density peak described earlier, points towards HOH 511 and forms a canonical hydrogen bond (O_w_…O_w_ distance: 2.82 Å, angle O_w_-H_w_…O_w_ = 136°). (ii) Its oxygen atom accepts a hydrogen bond from the NH2 group of the neighboring ASN_175 side chain (HD21…O_w_ distance: 2.23 Å, angle N-H…O_w_: 139°). (iii) The oxygen atom of HOH_524 is coordinated to the catalytic calcium (distance: 2.5 Å) and (iv) further interacts with the carboxylate side chain of Asp_229. The O_w_…Oδ2 and O_w_…Oδ1 distances are both 2.82 Å, which corresponds to standard hydrogen bond donor-acceptor distance in protein H…O bonds. More precisely, the location of the second hydrogen atom of HOH_524 between O_w_ and Asp_229 Oδ1 is less favorable, due to the vicinity of the calcium ion. Hence Oδ2 of Asp_229 is the only putative hydrogen bond acceptor atom for HOH_524. In this case, the O_W__511…O_W__524…Oδ2 angle is 120°, which fits perfectly with the possible hydrogen bond network involving both hydrogen atoms of HOH_524 water molecule. However, despite this ideal geometric configuration, the *F*_obs_-*F*_calc_ density map does not yield significant density peak for a second HOH_524 hydrogen atom interacting with Asp_229.

## Discussion

### Discrepancies between X-N and X-ray structures

The hydrogen bond network of water molecules is different in the X-N and X-ray structures, despite the similar localization of water oxygen atoms. In addition, the donor-acceptor (H…O) distances turn out to be shorter in the X-ray structure than in the X-N model. Such discrepancies are interesting and could be due to an intrinsic difference within the information from X-rays or neutrons, to different quality and resolution of the diffraction data sets or to the experimental setups, such as the differences in thermal motion for the water molecules between the two structures (see Additional file 1: Table S1), and the data collection temperature (cryogenic for X-rays, room temperature for neutrons). The last discrepancy is likely to affect the hydration pattern of proteins and the observed differences in solvation may reflect the effect of temperature.

### Active site calcium-bound water molecule

As underscored so far, the case of the calcium coordinated water molecule HOH_524 proves indeed noteworthy. First, because of its location close to Asp_229 and its coordination to the catalytic calcium. Secondly, because one of its hydrogen atoms is not clearly visible in the electronic density maps, although the oxygen atom is well confined by numerous interactions. This coordination is very different from the chemically unfavourable coordination of this water molecule in the X-N structure (Ca-O-H angle of 53° and Ca…H distance shorter than the van der Waals contact), that was attributed to the special environment of the protein active site [20].

Noteworthy, in the X-ray structure, this water molecule sits in a different orientation. As explained above, this observation may be the consequence of different experimental setups like temperature and consequently altered thermal motion parameters. Indeed, significant differences between structures at different temperature have sometimes been noted [21]. It is however interesting to notice that this conformation seems to be chemically more stable than that observed in the X-N structure. Indeed, the hydrogen atom of HOH-524, located with a strong electron density peak, points towards HOH 511 and forms a canonical hydrogen bond. A second interaction is observed with the carboxylate of Asp_229 (2.82 Å), but no significant electron density peak is visible in H omit maps above a 1 sigma level. However, a hydrogen atom could still be present: high atomic thermal motion, multiple conformations or diffuse position such as low-barrier H-bonds [19,22] can result in weak or even absent electron density for hydrogen atoms. Nevertheless, regarding the other interactions involving HOH-524, and especially the O_w_ … Asp_229_Oδ2 contact, one could expect both hydrogen atoms to be well defined, and their electron density peaks visible, similarly to the other water hydrogen atoms in the active site. A likely explanation involves a partially deprotonated HOH_524 water molecule. Indeed, Asp_229_Oδ2 may act as a base, accepting a proton from HOH_524, the latter being subsequently activated into a hydroxide ion. Such a mechanism is compatible with the nearby presence of the Ca^2+^, which acts as a Lewis acid by fostering the HOH_524 proton release (pK_a_ of H_2_O/ Ca^2+^ = 12.80 [23]) and interacting with the corresponding free electron doublet from the activated water molecule. In the framework of this mechanism, the observed electron density results from the occurrence of two protonation states of HOH_524, which may account for the weak electron density peak located between O_w__524 and Asp_229_Oδ2. The elliptical shape of the second hydrogen atom of HOH_524 electron density peak further corroborates such a statement inasmuch as it could reflect a superimposition of the hydrogen atom electron density in both HOH_524 protonation states. The two different states of the water molecule should adopt two slightly different orientations (Figure 4). Thus, findings heretofore suggest that this water molecule is partially deprotonated, with a hydrogen atom in a diffuse position. This observation contrasts with the X-N structure, where the corresponding water molecule is described as a classical H_2_O molecule [24].

### May the observable partially activated water molecule be involved in the DFPase catalytic mechanism?

The initial mechanism that was proposed for this enzyme consisted in a nucleophilic attack on the phosphorous center of the substrate by an activated hydroxide ion [10]. This mechanism is similar to those proposed for other organophosphorous hydrolases, such as the PTEs and PLLs [6,25], and the close DFPase structural homologues, the human paraoxonase [14] as well as the lactonase Drp35 [15]. Later on, however, while resorting to H_2_^18^O isotope labeling, the calcium-coordinated Asp_229 was thought to act as the nucleophile that attacks the phosphorus center of the substrate, via the formation of a phosphoenzyme intermediate [12]. This new mechanism, along with other reports such as stereospecificity studies [26], discarded the previous requirement for an activated water molecule. Noteworthy, the X-N structure of DFPase did not reveal the presence of an activated water molecule in the active site [24]. However, if converging elements suggests that Asp_229 is the nucleophile that attacks the phosphorus center, an important issue regarding enzyme regeneration, by a solvent water molecule, remains poorly addressed [12]. Could this observed partially activated water molecule play a role in the enzyme regeneration?

Several observations might speak in favor of this hypothesis. Firstly, a use of this water molecule during catalysis would be consistent with the previous observation that the binding of a substrate mimic to the DFPase yields a completely buried active site [12]. Secondly, the catalytic calcium coordination is plastic in DFPase [27], but also in structurally-related enzymes such as rHPON [14] and the gluconolactonase [28], a key property that may be utilized to reorganize the calcium coordination shell during catalysis. As an illustration, Glu_48 (in gluconolactonase structure, equivalent to Glu_21 for DFPase) adopts the same configuration as elicited in rHPON and DFPase structures: it is bound to the catalytic calcium cation. However, the second monomer of the asymmetric unit, Glu_21 does not interact directly with the catalytic calcium as in DFPase, but only indirectly, performing thereby a short hydrogen bond with a metal-bound water (see Additional file 1: Figure S1). Through a structural comparison between the DFPase, the rHPON and the gluconolactonase, the movement of Glu_21 that occurs in the gluconolactonase seems possible. The important plasticity of these active sites might allow a pre-existing water molecule to be re-located while the substrate binds to the active site. Noteworthy, HOH_524 in DFPase structure sits in a similar location than that of the proposed attacking water molecule in PON1 [14]: it is located in the vicinity of the catalytic calcium and interacts with Asp_269 that co-activates it (Asp_229 in DFPase). Altogether, the presence of a partially activated water molecule in the active site of the sub-Ångstrom resolution structure of DFPase might constitute an interesting piece of information, possibly useful to refine the DFPase mechanism.

## Conclusion

The analysis of the high-resolution X-ray structure of DFPase exemplifies that high-resolution X-ray crystallography can be an efficient technique to study hydrogen atoms location, when the data are appropriately refined. Our Sub-Ångstrom X-ray model enabled us to identify numerous water hydrogen atoms and to construct experimentally,--on the sole basis of electron density peaks--, the water hydrogen bonds network in the protein central tunnel. Thorough scrutiny of these H bonds networks in both our model and the available neutron X-N structure reveals different solvation states that may be due to the thermal motion or to experimental setups (e.g. temperature of data collection). They possibly reflect different states existing in solution. Our investigation into the DFPase active site in the X-ray structure suggests that the calcium-coordinated water molecule HOH_524 is partially activated into a hydroxide ion through proton relocation towards the nearby Asp_229 Oδ2 atom.

## Methods

### X-ray structure

The starting molecular file in SHELXL format [29] of the 0.85 Å resolution X-ray structure and the reflection file from an earlier study [11,17] were kindly provided by Koepke and colleagues. We performed an additional refinement of the DFPase model using *MoPro* software [30]. Among its distinctive features, MoPro software enables electron density refinement based on a non-spherical atom model [31], benefiting additionally from the aspherical scattering factor library ELMAM [32]. In the case of DFPase, the resolution of the diffraction data remains inadequate to perform satisfactorily the refinement of the multipolar parameters. Compared to the independent atom model, the number of parameters increases dramatically (from 10 up to 36 par atom) and a sizeable number of observations is required. Hence, the multipolar refinement is often performed locally in low thermal motion protein regions. The isotropic thermal displacement parameters *B*_eq_ of the atoms in the active site of DFPase are rather low for a protein (*B*_eq_ of residues in the active site: 5.3 Å^2^), however not low enough to reach effective multipolar refinement. Test refinements merely yielded unsatisfactory electron density parameters. Therefore, the scaling factor and solvent parameters (using exponential scaling model [33]) were refined. The quality of electron density maps enabled us to identify density peaks corresponding to some water hydrogen atoms in *F*_obs_-*F*_calc_ residual maps that were placed manually using the *Coot* program [34]. Proper angle and distance optimization was carried out using the *MoPro* software so as to comply with both the electron density peaks and the usual stereochemistry of the water molecule. The H_w_-O_w_-H_w_ angle for water was constrained at 104.5°. The O_w_-H_w_ bond length in water molecules observed in X-ray structures usually reaches 0.81 Å [35]. However, in order to compare the H-bond network in both the X-ray and X-N structures, the bond length has been elongated to the average value observed by neutron diffraction, namely 0.96 Å [35]. Such a discrepancy occurs as the electron of the hydrogen atom is shared within the covalent bond. Consequently, the X-H distances appear shorter in the X-ray than in the X-N structure. The ordered water molecules are mainly located in the water-filled tunnel of the protein and within the DFPase active site. The structure was deposited in the PDB (id:3O4P).

### X-N structure

The model and nuclear structure factors were retrieved from the Protein Data Bank (PDB [36] id: 3BYC). Structure factor phases for the nuclear density maps computation were obtained after a single refinement cycle (scale factor and solvent parameters) with the *Phenix* software v.1-4.138 [37]. The applied resolution, weighting schemes and diffraction data cut-offs were the same as those used in the reported DFPase X-N structure [20].

### Analysis of water hydrogen bond geometry

The geometric parameters of the hydrogen bond network formed by the water molecules in the X-ray and X-N structures were analyzed using *MoPro* software. Only H_w_…O hydrogen bonds were investigated, inasmuch as H_w_ is a water hydrogen atom and O a protein or water oxygen atom. Neighboring atoms which fulfilled required criteria were considered as hydrogen-bonded, i.e. whenever the H_w_…O distance was below 2.5 Å and the O_w_H_w_ …O angle above 90°. In the X-ray structure, the analysis was performed on the water molecules for which the H atoms were placed according to electron density. In the X-N structure, we analyzed the water molecules identified at positions equivalent to those in the X-ray structure. Based on the here-above mentioned criteria for H bonding, thirty and thirty-eight hydrogen bonds were considered for the X-ray and X-N structures, respectively.

## Competing interests

The authors declare no conflict of interests.

## Authors’ contributions

DL, JK processed the data; DL refined the structure; DL, ME, CL, BG, CJ, EC analyzed the data; ME, DL, CJ, EC wrote the manuscript. All authors read and approved the final manuscript.

## Supplementary Material

Additional file 1 **B factor analysis of water oxygen atoms (Additional file ****1****: Table S1) and close view within the active site of the gluconolactonase (Additional file ****1****: Figure S1).** Click here for file
