# Supplementary material for: Hydrogen atoms in protein structures: high-resolution X-ray diffraction structure of the DFPase
Source: BMC Res Notes. 2013 Aug 2;6:308. doi: 10.1186/1756-0500-6-308 (PMC3737025; doi:10.1186/1756-0500-6-308)
Supplement: Additional file 1 — B factor analysis of water oxygen atoms (Additional file 1: Table S1) and close view within the active site of the gluconolactonase (Additional file 1: Figure S1). [file 1756-0500-6-308-S1.docx]

**SUPPLEMENTARY INFORMATION**

| **X-rays** | | **X-N** | | **subset** |
| --- | --- | --- | --- | --- |
| **HOH** | ***B*_eq_ [Å^2^]** | **DOD** | ***B*_eq_ [Å^2^]** |  |
| 509 | 6,3 | 1052 | 15,2 | 1  (different orientations) |
| 511 | 7,8 | 1010 | 45,0 |  |
| 604 | 5,4 | 1034 | 18,3 |  |
| 524 | 6,9 | 1033 | 26,0 |  |
| 703 | 7,9 | 1066 | 24,2 |  |
| 613 | 8,8 | 1089 | 23,7 |  |
| 607 | 7,2 | 1016 | 23,9 |  |
| 505 | 6,1 | 1095 | 22,0 |  |
| 543 | 7,2 | 1057 | 23,0 |  |
| 705 | 10,0 | 1096 | 33,7 |  |
| 538 | 8,9 | 1064 | 29,6 |  |
| **means** | 7.5 (sd : 1.4) |  | 25.9 (sd : 8.0) |  |
| 683 | 7,1 | 1054 | 24,1 | 2  (similar orientations) |
| 527 | 7,6 | 1013 | 51,9 |  |
| 502 | 5,6 | 1032 | 15,7 |  |
| 501 | 5,1 | 1050 | 14,4 |  |
| 623 | 5,5 | 1055 | 26,9 |  |
| 615 | 5,8 | 1015 | 14,9 |  |
| 614 | 5.7 | 1036 | 22,7 |  |
| 580 | 7,9 | 1076 | 32,8 |  |
| 512 | 6,9 | 1037 | 25,7 |  |
| **means** | 6.4 (sd: 1.1) |  | 25.5 (sd : 11.7) |  |
| **HOH total** | 481 |  | - |  |

**Table S1**: Equivalent isotropic *B*_eq_ factor of water oxygen atoms, located at similar positions in the X-ray and neutron structures. sd = standard deviation.

**Figure S1**


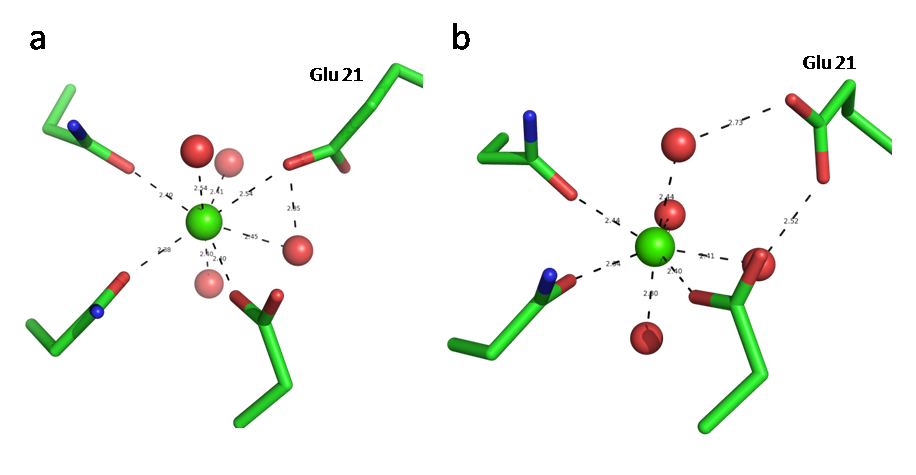


**Figure S1:** Close view within the active site of the gluoconolactonase. (a) The active site configuration in the monomer A of the gluconolactonase, where Glu 21 adopts a similar configuration to that observed in the DFPase and rHPON structures. (b) Active site configuration in the monomer B of the gluconolactonase, where Glu 21 rotates and interacts with the catalytic calcium (green sphere) ion via a water molecule (red sphere).
